# Mechanisms of Resistance to Spot Blotch in Yunnan Iron Shell Wheat Based on Metabolome and Transcriptomics

**DOI:** 10.3390/ijms23095184

**Published:** 2022-05-06

**Authors:** Xuesong Zhang, Tingzhi Huang, Qianchao Wang, Yirui Guo, Ping Zhang, Heng Xie, Junna Liu, Li Li, Chuanli Zhang, Peng Qin

**Affiliations:** 1 College of Agronomy and Biotechnology, Yunnan Agricultural University, Kunming 650201, China; 2020240160@stu.ynau.edu.cn (X.Z.); 2020240162@stu.ynau.edu.cn (T.H.); 2020110028@stu.ynau.edu.cn (Q.W.); 2020240137@stu.ynau.edu.cn (Y.G.); 2021110031@stu.ynau.edu.cn (P.Z.); 2020210159@stu.ynau.edu.cn (H.X.); 2021110026@stu.ynau.edu.cn (J.L.); 2019210130@stu.ynau.edu.cn (L.L.); 2 College of Tropical Crops, Yunnan Agricultural University, Pu’er 665000, China

**Keywords:** *Triticum aestivum* ssp., spot blotch, metabolome, transcriptome, benzoxazinoid biosynthesis

## Abstract

Spot blotch (SB) is a fungal disease that threatens wheat yield and quality. Presently, the molecular mechanism against SB is unclear. In this study, the resistant variety Zhenkang iron shell wheat (Yunmai 0030) and susceptible variety Lincang iron shell wheat (Yunmai 0608) were selected by identifying SB of Yunnan iron shell wheat. The metabolome and transcriptome of leaves of two varieties at different positions were detected using the systemic acquired resistance theory to investigate the molecular and physiological changes in Yunnan iron shell wheat under SB stress. We found that the genes and metabolites related to benzoxazinoid biosynthesis and arginine and proline metabolism were highly enriched after infection with leaf blight. The enriched differential metabolites mainly included phenolic acids, alkaloids, and flavonoids. We further observed that DIBOA- and DIMBOA-glucoside positively affected iron shell wheat resistance to leaf blight and proline and its derivatives were important for plant self-defense. Furthermore, we confirmed that the related metabolites in benzoxazinoid biosynthesis and arginine and proline metabolism positively affected *Triticum aestivum* ssp. resistance to SB. This study provides new insights into the dynamic physiological changes of wheat in response to SB, helps us better understand the mechanism of resistance to SB, and contributes to the breeding and utilization of resistant varieties.

## 1. Introduction

*Triticum aestivum* ssp. *yunnanense* King is a unique hexaploid wheat germplasm resource in Yunnan, China and is one of the three common wheat subspecies unique to China [1,2,3]. It is mainly planted in the Lincang, Baoshan, and Simao areas [4,5]. With excellent agronomic traits, rich genetic variation, and good disease resistance, *T. aestivum* ssp. is an important genetic resource for breeding new wheat disease-resistant varieties and a bridge germplasm resource for exploring the origin and evolution of cultivated hexaploid wheat.

Spot blotch (SB), caused by *Biopolaris sorokiniana*, is a devastating disease of wheat [6] that generally occurs upward from the old leaves at the base of the plant. It is difficult to control SB effectively, owing to its strong genetic diversity and regular sexual recombination in the wheat-growing season [7,8,9,10]. The disease severity is affected by management, soil, planting density, and weather [11]. The occurrence of SB seriously affects the normal growth and development of wheat, leads to early wheat maturity, and seriously affects the quality of grain [12,13,14]. Recently, SB occurrence has become increasingly frequent and the coverage areas and degrees of damage have been expanding [15,16]. The scope of occurrence in China has spread from frequent areas in the northeast and northwest to China’s main wheat-producing areas in the middle and lower reaches of the Yangtze River, Huang Huai Hai, and the southwest; the highest grain loss can be as high as 50% [17,18]. Globally, the occurrence of SB covers wheat-growing areas worldwide, such as the Middle East, Europe, North America, South America, Australia, and Southeast Asian countries [19,20,21,22]. SB has evolved resistance to almost all fungicides, including azole fungicides and succinate dehydrogenase inhibitors [23]; therefore, it is urgent to study the wheat’s relevant resistance mechanism to SB and cultivate SB-resistant varieties.

Plants possess a defense mechanism that is induced after being infected by pathogens. This defense is usually not against a single pathogen but a wide range of microorganisms. Therefore, different plants have evolved the same defense effect and have broad-spectrum resistance to pathogens [24,25]. In a recent study, Sebold et al., proved for the first time that systemic immune signals can improve the resistance of plant system tissues to pathogens. Immune signals from infected sites spread to other sites to resist pathogen infection and this phenomenon is called systemic induced sensitivity [26]. As the final product of the cell regulation process, the expression level of metabolites can be regarded as the final response of the biological system to genetic or environmental changes. They directly participate in the response of plants to the external environment and affect or regulate gene transcription, protein expression, and activity [27]. For example, chitinase produced after plant infection by pathogenic bacteria is an anti-fungal protein that inhibits spore germination and the mycelial growth of pathogenic fungi to activate the defense genes in the entire plant, developing plant resistance to biological stress. Flavonoid, a secondary metabolite, can kill fungal pathogens that invade plants and terpenoids can kill various human invasive eubacterial and viral pathogens [28].

In addition, Benzoxazinoid class compounds (DIBOA-glucoside and DIMBOA-glucoside), mainly synthesized by gramineous plants, play an important role in resistance against biological and abiotic stresses. Therefore, the improvement of the economic importance of SB is closely related to the improvement of wheat varieties and the promotion of improved varieties. Some studies have found evidence that links genetic resistance to SB with multiple effector genes [29]. Presently, only four SB resistance genes, namely *Sb1* [30], *Sb2* [31], *Sb3* [32], and *Sb4* [33], have been reported. In this study, we analyzed the correlation between different metabolites and genes among susceptible and resistant strains and the resistance mechanism of iron shell wheat to leaf blight by evaluating the disease resistance of *Triticum aestivum* ssp. *yunnanense*, a unique wheat resource in Yunnan, and excavating excellent disease resistance genes. We also analyzed the metabolic pathway or network of *Triticum aestivum* infected with SB to reveal the response mechanism of relevant metabolites and genes and to explore the resistance mechanism of *Triticum aestivum* to SB, thereby providing an important reference for broadening the cultivation, development, and utilization of resistant wheat varieties.

## 2. Results

### 2.1. Detection Results and Analysis of Widely Targeted Metabolome

#### 2.1.1. Quality Control of Widely Targeted Metabolome Data

**Figure 1:**
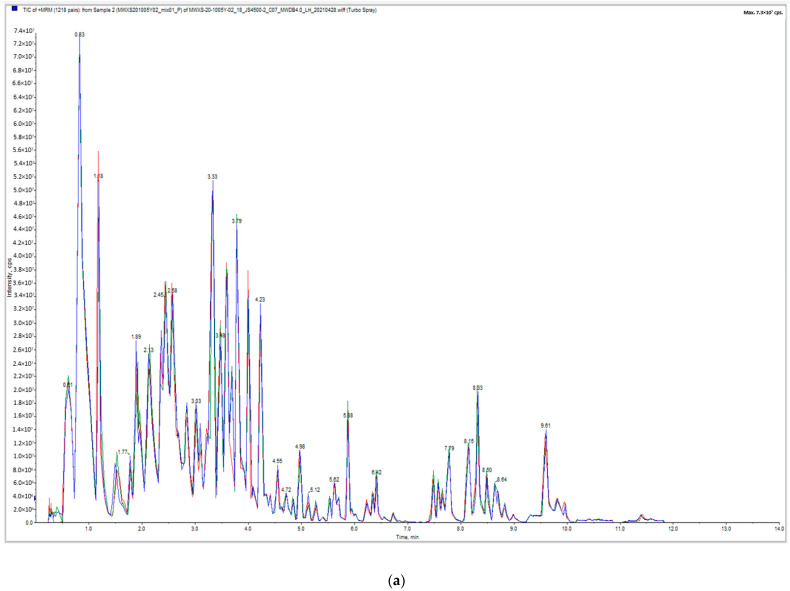

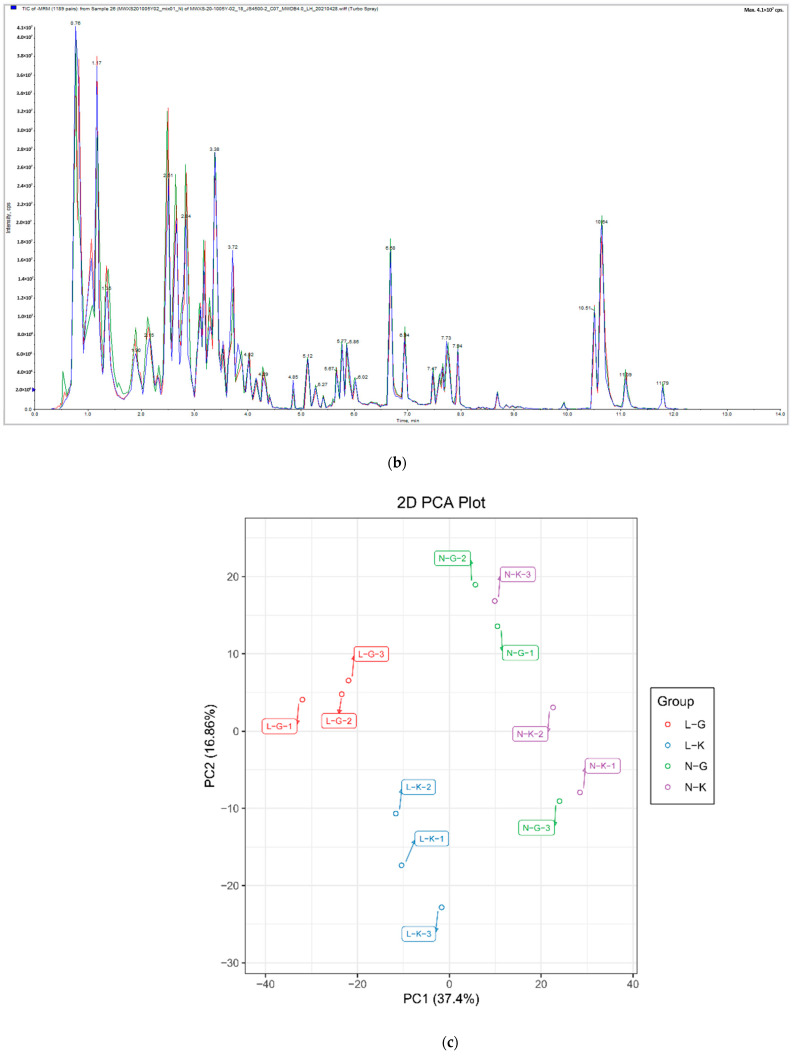

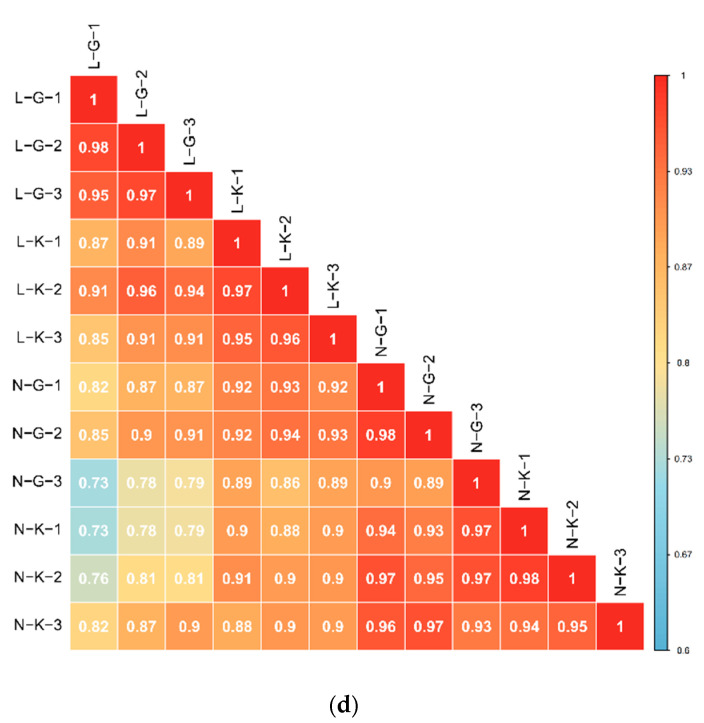
Tic overlap diagram of quality control (QC) sample mass spectrometry detection: (**a**) positive ion mode, (**b**) negative ion mode, (**c**) principal component analysis (PCA) score diagram of all samples, and (**d**) correlation diagram between samples. Note: PC1 represents the first principal component, PC2 represents the second principal component, and the percentage represents the interpretation rate of the principal component to the data set; each point in the figure represents a sample, and the samples of the same group are represented by the same color.

The overlapping display analysis of the total ion flow detected and analyzed by mass spectrometry of quality control samples (QC) revealed high overlap in positive and negative ion modes (Figure 1a,b). From the figures, it can be observed that the experimental technology had high repeatability and overlap, providing a guarantee for the authenticity and reliability of the data. According to the PCA score Figure 1c) and sample correlation diagrams (Figure 1d), the Pearson correlation coefficient in the four groups of samples was r > 0.8, with good overall repeatability and high intragroup correlation. In the PCA analysis, PC1 and PC2 were 34.67% and 15.23%, respectively, and significant differences in metabolites were observed among groups, with L-G and N-G being the most apparent. The metabolome data in this study met the research needs and are true and reliable.

#### 2.1.2. Qualitative and Quantitative Analysis of Metabolome

The metabolites at two positions of the two strains were detected qualitatively and quantitatively by the widely targeted metabolome determination method. The results showed that (Table 1) 1078 metabolites were detected, including 95 amino acids and their derivatives, 178 phenolic acids, 67 nucleotides and their derivatives, 234 flavonoids, 30 lignans and coumarins, 3 blends, 111 alkaloids, 22 terpenoids, 84 organic acids, 154 lipids, and 100 others. Furthermore, from the clustered heat map of sample metabolites Supplementary Figure S1), it was observed that there were significant differences in metabolite contents between susceptible and resistant strains and between diseased and normal parts of the same plant. Furthermore, before the differential analysis, the orthogonal partial least squares discriminant analysis (OPLS-DA) score diagram (Supplementary Figure S2) and OPLS-DAs-da verification diagram (Supplementary Figure S3) of samples from each group showed significant differences between the groups, especially comparing the same part of the susceptible and resistant strains. Q2 was greater than 0.85 and most of the *p*-values of the model were less than 0.05, indicating that the prediction ability of the model was better.

#### 2.1.3. Differential Analysis of Metabolic Profiles between Resistant and Susceptible Strains

**Figure 2:**
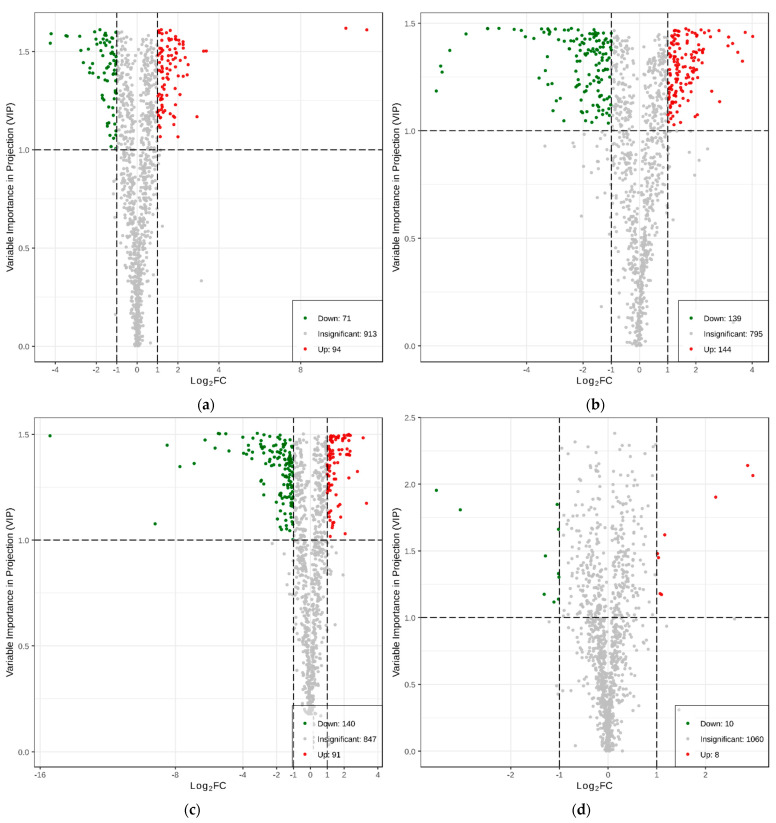

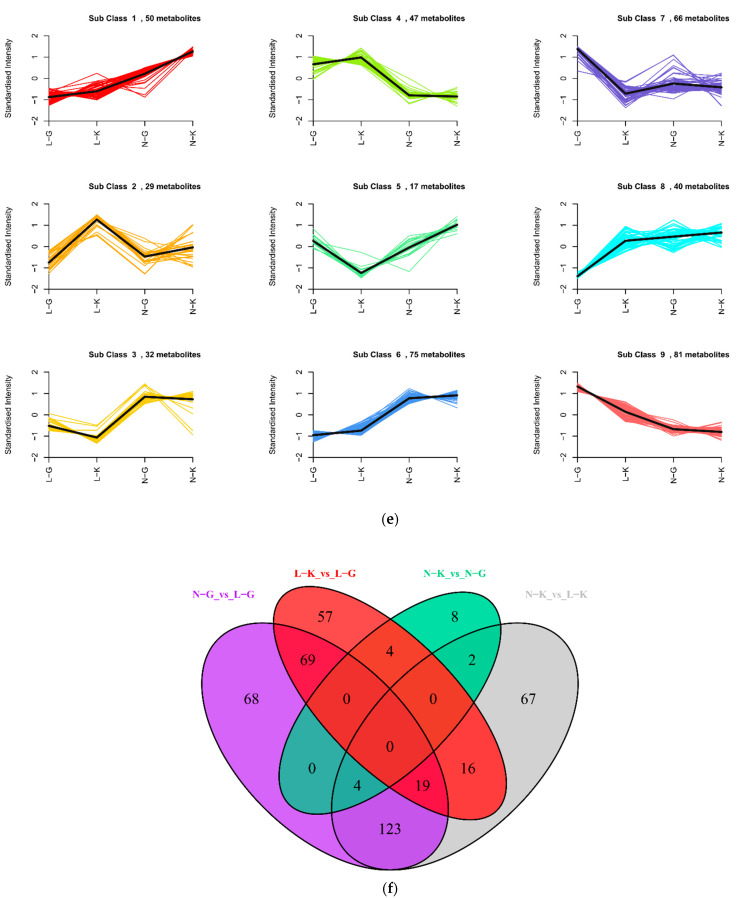
Differential metabolite volcano maps: (**a**) L-K vs. L-G, (**b**) N-G vs. L-G, (**c**) N-K vs. L-K, and (**d**) N-K vs. N-G. Each point in the volcano map represents a metabolite, in which the green points represent downregulated differential metabolites, the red points represent upregulated differential metabolites, and the gray points represent the detected but insignificant metabolites. The abscissa represents the logarithm (log2FC) of the quantitative difference multiples of a metabolite in two samples. The greater the absolute value of the abscissa the greater the differential expression. (**e**) Differental metabolite K-means diagram, (**f**) Venn diagram, wherein each circle represents a comparison group. The number of circles and overlapping parts represents the number of common differential metabolites between the comparison groups and the number without overlapping parts represents the number of unique differential metabolites in the comparison group.

Based on the OPLS-DA results, the variable importance projection (VIP) of the OPLS-DA model was analyzed using multiple variables, and the metabolites with fold change ≥ 2 or ≤0.5 and VIP ≥ 1 were selected as the metabolites to be compared between groups. Consequently, a total of 437 differential metabolites were detected. The volcanic maps of differential metabolites among the groups (Figure 2a–d) showed the significance of differential expressions between groups. The results showed that there were 165 differential metabolites (94 upregulated and 71 downregulated) between L-K and L-G; 283 differential metabolites (144 upregulated, 139 downregulated) between N-G and L-G; 231 differential metabolites (91 upregulated, 140 downregulated) between N-K and L-K; and 18 differential metabolites (8 upregulated, 10 downregulated) between N-K and N-G.

To study Triticale’s resistance to SB, we focused on differential metabolites as they related to L-K vs. L-G, N-G vs. L-G, and N-K vs. L-K. The top 20 differential metabolites among the groups are shown in Supplementary Figure S4. Among the 165 differential metabolites between L-K and L-G, the first three were phenolic acids, alkaloids, and flavonoids. The two most significantly upregulated differential metabolites were L-cysteine and acetate, with log2fc values of 11.2 and 10.2, respectively; nine of the ten most significantly downregulated differential metabolites were phenolic acids, including: 1,3-O-di-p-coumaroylglycerol, 1-O-feruloyl-3-Op-coumaroylglycerol, and 1-feruloyl-sn-glycerol. Among the 283 differential metabolites in the N-G vs. L-G combination, flavonoids, alkaloids, and phenolic acids showed the most significant differences. The two most significantly upregulated metabolites were 3,4-dihydroxy-L-phenylalanine (levodopa) and 7-O-methylnaringenin, and the most significantly downregulated metabolite was Coix (MBOA). In addition, three terpenoids—27,28-dicarboxyl ursolic acid, asiatic acid, and geniposide—were detected in L-K vs. L-G and N-G vs. L-G. Among the 231 differential metabolites of N-K vs. L-K were upregulated substances such as 3-indolepropionic acid and L-tryptophan and downregulated substances such as acetate (2-oxo-3h-1,3-benzoxazol-6-y1), L-cysteine, MBOA, and DIMBOA; most of the differential metabolites belonged to flavonoids, alkaloids, and phenolic acids. Eight of the top ten metabolites downregulated in N-G vs. L-G and N-K vs. L-K are similar.

The Venn diagram (Figure 2f) demonstrates that L-K vs. L-G, N-G vs. L-G, and N-K vs. L-K had 57, 68, and 67 specific differential metabolites, respectively. Therefore, we speculated that the metabolites closely related to the resistance of *Triticum aestivum* to SB exist in 19 differential metabolites shared by the three combinations such as 4-acetylaminobutyric acid and 2,4-dihydroxy-1,4-benzoxazine-3(4H)-ketoglucoside (DIBOA glucoside). The relative contents of all differential metabolites identified according to the screening criteria in all grouping comparisons were standardized by the Z-score and, further, the changed trend results of nine differential metabolites were obtained after K-means cluster analysis. The classification of metabolites in each cluster is shown in Figure 2e (Supplementary Table S1). The results showed that the contents of metabolites in clusters one, six, and eight were higher than those in L-G, indicating that metabolites such as DIMBOA and o-aminobenzoic acid showed a downward trend in susceptible plants when infected by SB. In contrast, the content of metabolites in cluster nine L-G was significantly higher than that in other groups and the content of metabolites showed an upward trend in susceptible plants such as indole and trans-4-hydroxy-l-proline*.

#### 2.1.4. Enrichment Analysis of Differential Metabolite KEGG Pathway after SB Infection

**Figure 3:**
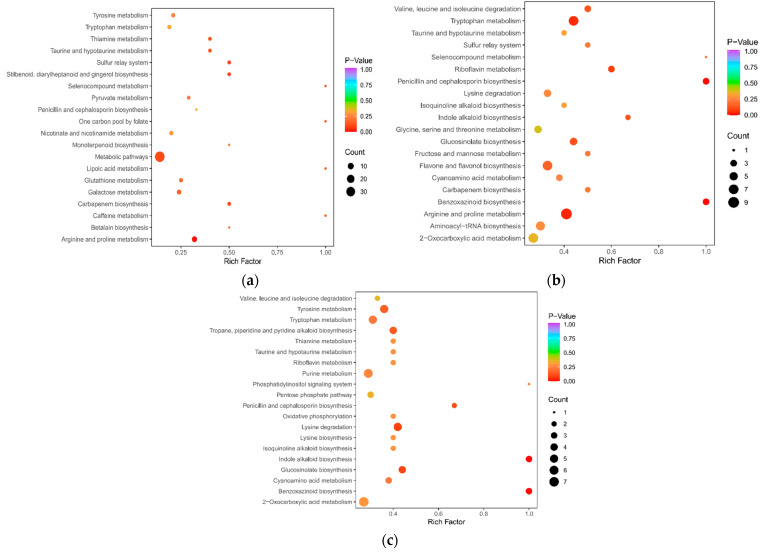
KEGG enrichment bubble. (**a**) L-K vs. L-G. (**b**) N-G vs. L-G. (**c**) N-K vs. L-K. The abscissa represents the Rich Factor corresponding to each pathway, and the ordinate represents the pathway name. The color of points reflects the *p*-value size, and the redder indicates the more significant enrichment. The size of the dot represents the number of enriched differential metabolites.

The enrichment analysis of the KEGG pathway was conducted according to the metabolic results of poor foreign bodies. If *p*-value ≤ 0.05, the pathway is enriched and the annotation results are significant differences. Figure 3 shows the first 20 pathways enriched in the three combinations. A total of 54 metabolic channels were enriched in L-K vs. L-G, of which only arginine and proline metabolism were significantly different. Seven metabolites (three organic acids and four phenolic amines) were enriched in this pathway. Metabolites such as agmatine, p-coumaroylagmatine and N-feruloylagmatine, N-acetylputrescine γ- organic acids, and 4-guanidine butyric acid were significantly upregulated, while 4-acetylaminobutyric acid was significantly downregulated. N-G vs. L-G involved a total of 63 metabolic pathways, including 4 metabolic pathways, namely penicillin and cephalosporin biosynthesis, benzoxazinoid biosynthesis, arginine and proline metabolism, and tryptophan metabolism, with significant differences. The nine metabolites involved in arginine and proline metabolism were upregulated, except γ- aminobutyric acid, which had five other upregulated metabolites, similar to those in L-K vs. L-G, and four metabolites, p-coumaroylputrescine, n-feroylputrescine, 4-acetamidobutyric acid, and trans-4-hydroxy-l-proline*. The metabolites that changed in this pathway mainly comprised alkaloids and organic acids. Three different metabolites were enriched in the biosynthesis of benzoxazocine; indole was upregulated and DIBOA and DIMBOA were downregulated. N-k vs. L-K co-annotation enriched 71 metabolic pathways, including 2 with *p*-value ≤ 0.05, namely indole alkaloid biosynthesis and benzoxazinone biosynthesis.

### 2.2. Transcriptome Test Results and Analysis

#### 2.2.1. Assembly Notes

**Figure 4:**
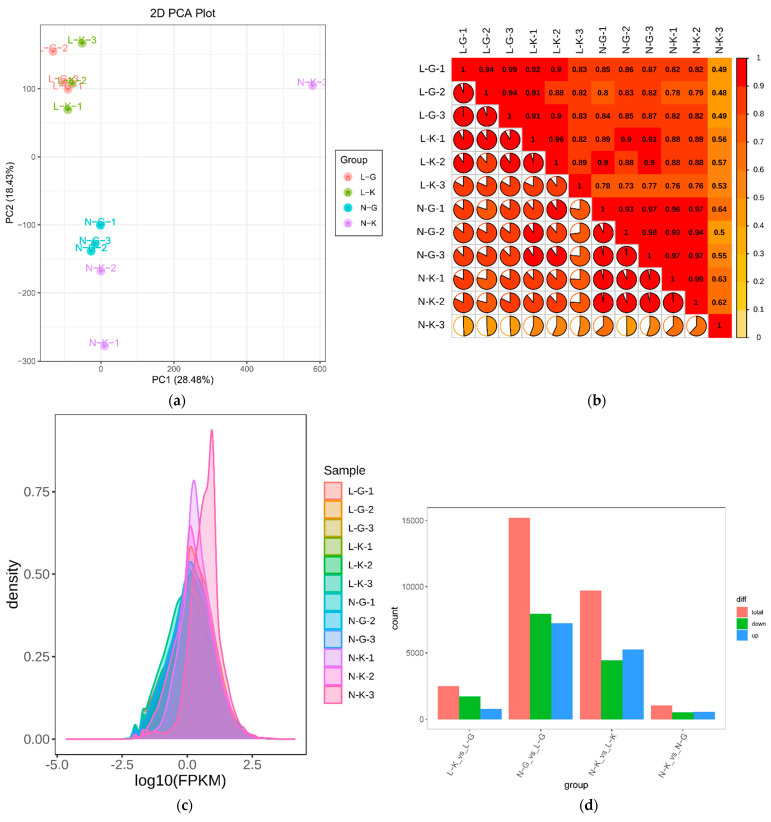

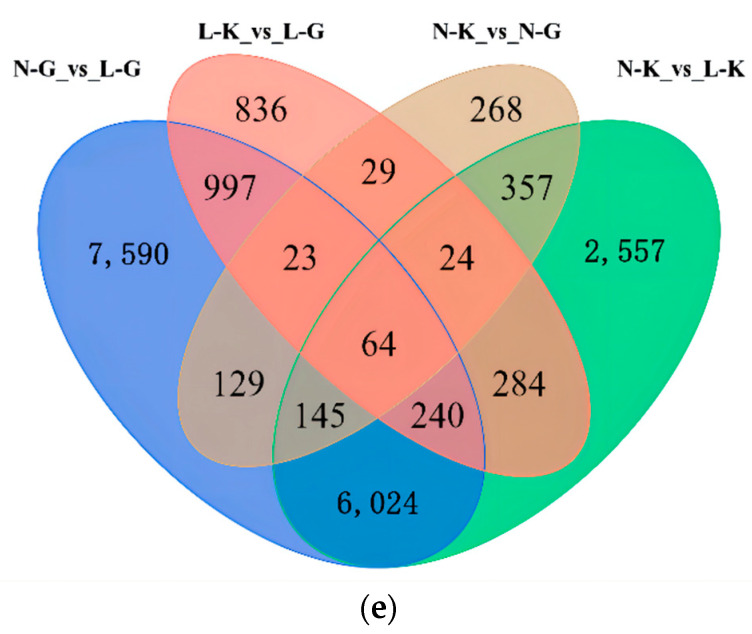
Note: in (**a**), PCA diagram, PC1 represents the first principal component, PC2 represents the second principal component, and the percentage represents the interpretation rate of the principal component to the data set; each point in the figure represents a sample, and the samples of the same group are represented by the same color. In the (**b**), Correlation heat map, r^2^ is to 1, the stronger the correlation between the two repeated samples is. Curves in different colors in (**c**) represent different samples. Expression density distribution. The abscissa of points on the curve represents the log value of the FPKM of the corresponding sample, and the ordinate of points represents the probability density. (**d**) represents the number of differentially down-regulated genes in different combinations. Differential gene histogram. In the Venn diagram (**e**), Venn diagram, the non-overlapping region represents the unique differential genes of the differential group and the overlapping region represents the common differential genes of several overlapping differential groups.

RNA-seq was performed on four groups of samples. Clean reads (163.17 GB) were obtained and the clean data of each sample reached 12 GB. The error rate (overall sequencing error rate), Q20, and Q30 values were <0.03%, >97%, and >93%, respectively. The average proportion of GC was 53.23%. The overall data show that the sequencing quality is high, which meets the requirements of the next analysis. The correlation analysis of gene expression levels between samples shows that the samples have high similarity in the same group, ensuring the reliability of subsequent differential gene analysis. PCA analyzed the gene expression differences among 12 samples (Figure 4a), which showed that the significant difference between susceptible and uninfected plants was obtained from PC1 (28.48%), while the difference between varieties was primarily obtained from PC2 (18.43%). According to the correlation heat map between samples (Figure 4b), the Pearson correlation coefficient among L-K, L-G, and N-G samples was >0.8, indicating that the repeatability between samples was high and met the research requirements. The genes detected in this experiment were annotated in GO, KEGG, KOG, NR, Pfam, Swiss port, and tremble databases, and 97971, 69484, 102341, 131395, 7370, 81729, and 134881 genes were annotated, respectively.

The gene differences among the four comparison groups are shown in Figure 4c. A total of 2497 (1721 downregulated and 776 upregulated), 15212 (7962 downregulated and 7250 upregulated), 9695 (4430 downregulated and 5265 upregulated), and 1039 (498 downregulated and 541 upregulated) differential genes were observed in L-K vs. L-G, N-G vs. L-G, N-K vs. L-K, and N-K vs. N-G, respectively. The gene expression density map shows the change trend of gene abundance with the expression amount in 12 samples. The logarithm value that clearly reflects the gene expression amount (fpkm) in the sample is concentrated between −2 to 2 (Figure 4c).

The Venn diagram analysis (Figure 4e) shows the number of common differential and unique genes between different combinations. For example, there were 64 common differential genes in N-G vs. L-G, L-K vs. L-G, N-K -vs. L-K, and -N-K vs. N-G, of which 7950, 836, 2557, and 268 specific differential genes were observed in N-G vs. L-G, L-K vs. L-G, N-K vs. L-K, and N-k vs. N-G, respectively.

#### 2.2.2. Differential Genes and GO and KEGG Enrichment Analysis

**Figure 5:**
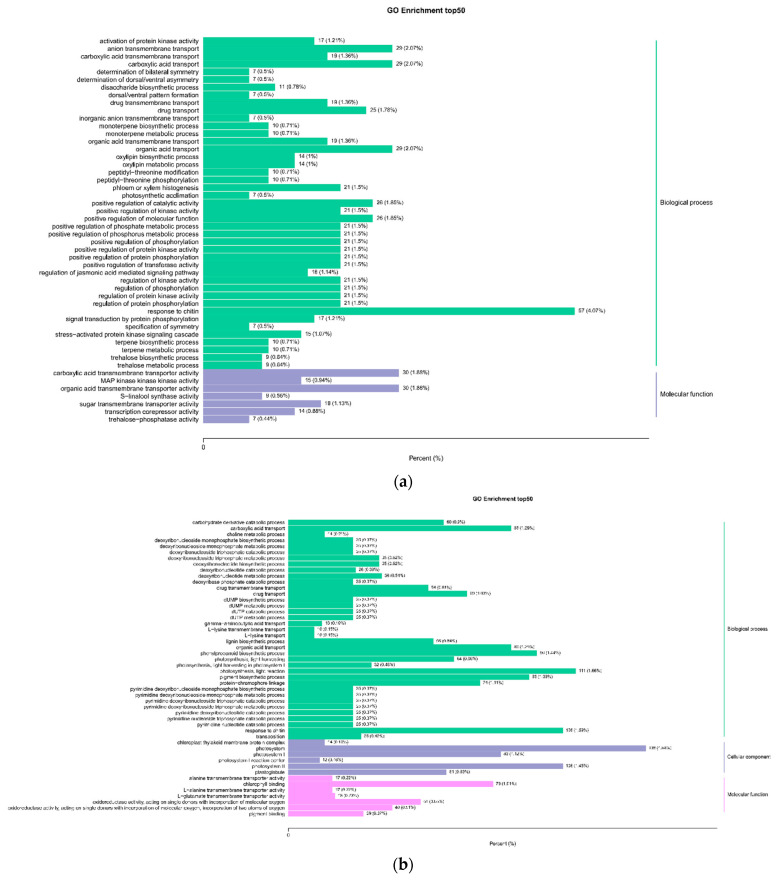

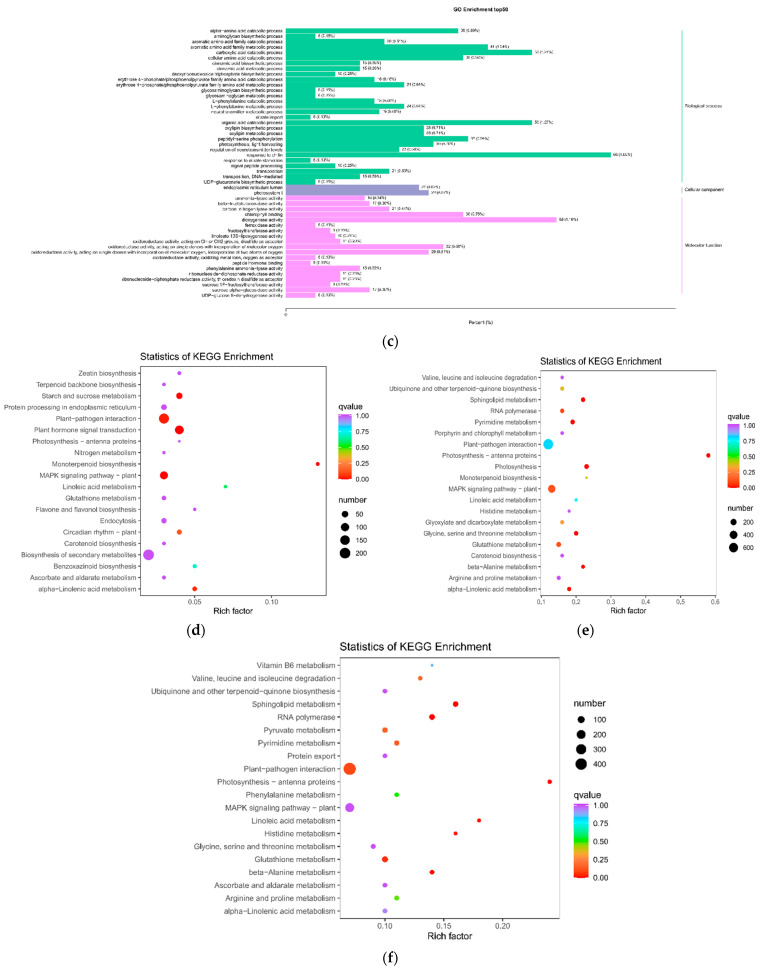
(**a**) L-K vs. L-G. (**b**) N-G vs. L-G. (**c**) N-K vs. L-K. (**d**) L-K vs. L-G. (**e**) N-G vs. L-G. (**f**) N-K vs. L-K. Histogram of GO enrichment entries (**a**–**c**) and KEGG enrichment scatter diagram (**d**–**f**).

The GO analysis of transcriptome showed 27 items related to biological processes, among which cellular process, metabolic process, response to stimulation, and biological regulation were the most important items. There were 18 items related to cell composition, among which GO items are most significantly enriched in cells, cell parts, organelles, and membranes; thirteen GO items related to molecular function were identified, of which, the contribution of binding and catalytic activity was the largest. GO enrichment and hierarchical analysis were carried out on the differential genes of each combination and 50 GO terms with the lowest Q value in the enrichment analysis results were selected to draw the column diagram of enrichment items (Figure 5a–c). The results showed that these differential genes were mainly enriched in photosynthesis (i.e., light system I\II, daylighting and light reaction), the biosynthesis process of phenylpropane, the activity of oxidoreductase with different functions, chlorophyll-binding, organic acid transport, signal transduction (i.e., kinase signal, phosphorylation), and plant hormone (jasmonic acid).

The KEGG enrichment analysis results showed that a total of 149 KEGG pathways were annotated, including 10, 29, 21, and 11 pathways that showed significant enrichment of L-K vs. L-G, N-G vs. L-G, N-K vs. L-K, and N-K vs. N-G, respectively. Among the L-K vs. L-G, N-G vs. L-G, and N-K vs. L-K groups, the pathways with the top 20 enrichment significances were selected and are shown in Figure 5d–f. The results showed that the most significant pathways in L-K vs. L-G were plant hormone signal transduction and monoterpene biosynthesis, MAPK signal pathway-plant, and plant-pathogen interaction pathways. The three pathways of photosynthetic antenna protein and photosynthesis and sphingolipid metabolism were the most significant in N-G vs. L-G. Sphingolipid metabolism and photosynthetic antenna protein were the most significant pathways in N-K vs. L-K. Six significantly different metabolic pathways—MAPK signaling pathway plant, plant pathogen interaction, monoterpene biosynthesis, α-linolenic acid metabolism, linoleic acid metabolism, and benzoxazine biosynthesis—were identified in L-K vs. L-G and N-G vs. L-G groups. L-K vs. L-G and N-K vs. N-G had the same three significantly different metabolic pathways: plant pathogen interaction, monoterpene biosynthesis, and MAPK signaling pathway. N-G vs. L-G and N-K vs. L-K had 15 identical and significantly different metabolic pathways, including photosynthetic antenna protein and RNA polymerase β- Alanine metabolism. In conclusion, the key metabolic pathways of *T. aestivum* ssp against SB are benzoxazinoid biosynthesis, arginine, and proline metabolism.

#### 2.2.3. Screening of Genes Related to Metabolic Pathway Differences

In Benzoxazine biosynthesis, 12 genes (9 upregulated and 3downregulated) and 37 genes (28 upregulated and 9 downregulated) were annotated in L-K vs. L-G and N-G vs. L-G groups, respectively. There were three upregulated genes in L-K vs. L-G and N-G vs. L-G, and among them, the enzyme corresponding to gene: *TraesCS6B02G158300* was indole-2-monooxygenase [EC: 1.14.14.153] (BX2). The enzyme corresponding to *gene:TraesCS3A02G06220* and *gene:TraesCS5B02G318200* was indole-2-one monooxygenase [EC: 1.14.14.157] (BX3). In the metabolism of arginine and proline, 8 genes were annotated in L-K vs. L-G, and all were downregulated, and 48 genes were annotated in N-G vs. L-G (19 upregulated and 29 downregulated). There were five similar genes in L-K vs. L-G and N-G vs. L-G, of which, the corresponding enzymes of *gene:TraesCS3A02G363700* and *gene:TraesCS3D02G357200* were glutamate 5-kinase [EC: 2.7.2.11] (proB) and glutamate -5-semialdehyde dehydrogenase [EC: 1.2.1.41] (proA), respectively. *Gene:TraesCS2B02G347800*, *gene:TraesCS5B02G220000*, and *novel.5994* correspond to agmatine deaminase [EC: 3.5.3.12] (augA), S-adenosylmethionine decarboxylase [EC: 4.1.1.50] (speD/AMD1), and aldehyde dehydrogenase (NAD +) [EC: 1.2.1.3] (ALDH), respectively.

#### 2.2.4. RT-qPCR Validation

**Figure 6:**
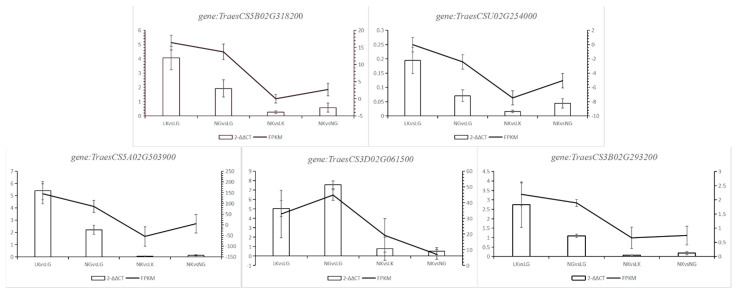
RT-qPCR Verification results.

To determine the authenticity and reliability of transcriptome data and the differential expression level of candidate genes, several genes were verified using RT-qPCR. The results showed that the RT-qPCR results of five genes (*gene:TraesCS5B02G318200*, *gene:TraesCSU02G254000*, *gene:TraesCS5A02G503900*, *gene:TraesCS3A02G061500*, and *gene:TraesCS3B02G293200*) were consistent with RNA SEP data. The specific results are shown in Figure 6.

### 2.3. Combined Analysis of Transcription and Metabolome

#### 2.3.1. Connection between DEGs and Dams

To understand the relevant mechanism of SB resistance of *Triticum aestivum*, metabolomics and transcriptomics data were integrated and analyzed. KEGG enrichment analysis *p*-value histogram (Supplementary Figure S5) showed that the differential metabolites and genes of resistant and susceptible strains were significantly enriched only in the “benzoxazinone biosynthesis pathway” and “arginine and proline metabolism pathway” (*p* < 0.05). It was further confirmed that the resistance of Triticale to SB was regulated by DEGs and DAMs related to these two pathways.

We analyzed the co-expression network of transcriptome and metabolome to further study the relationship between DEGs and DAMs after being infected with SB. The nine-quadrant diagram (Supplementary Figure S6) showed the differential multiple of gene metabolites with a Pearson correlation coefficient > 0.8 in each differential group, and the genes and metabolites in quadrants three and seven showed the same differential expression pattern, showing that these metabolites were positively regulated by genes. The differential expression pattern of genes in quadrants one and nine was opposite to that of metabolites and these metabolites are negatively regulated by genes. For example, in L-K vs. L-G, 3279 genes positively regulated 173 metabolites and 3382 genes negatively regulated 174 metabolites. N-G vs. L-G consisted of 10902 genes positively regulating 315 metabolites and 13069 genes negatively regulating 768 metabolites. N-K vs. L-K consisted of 15607 genes positively regulating 244 metabolites and 12483 genes negatively regulating 247 metabolites. N-K vs. N-G had 1812 genes that positively regulated 19 metabolites and 1048 genes that negatively regulated 21 metabolites.

#### 2.3.2. Correlation Analysis of Differential Genes and Metabolites in “Benzoxazinoid Biosynthesis” and “Arginine and Proline Metabolism”

**Figure 7:**
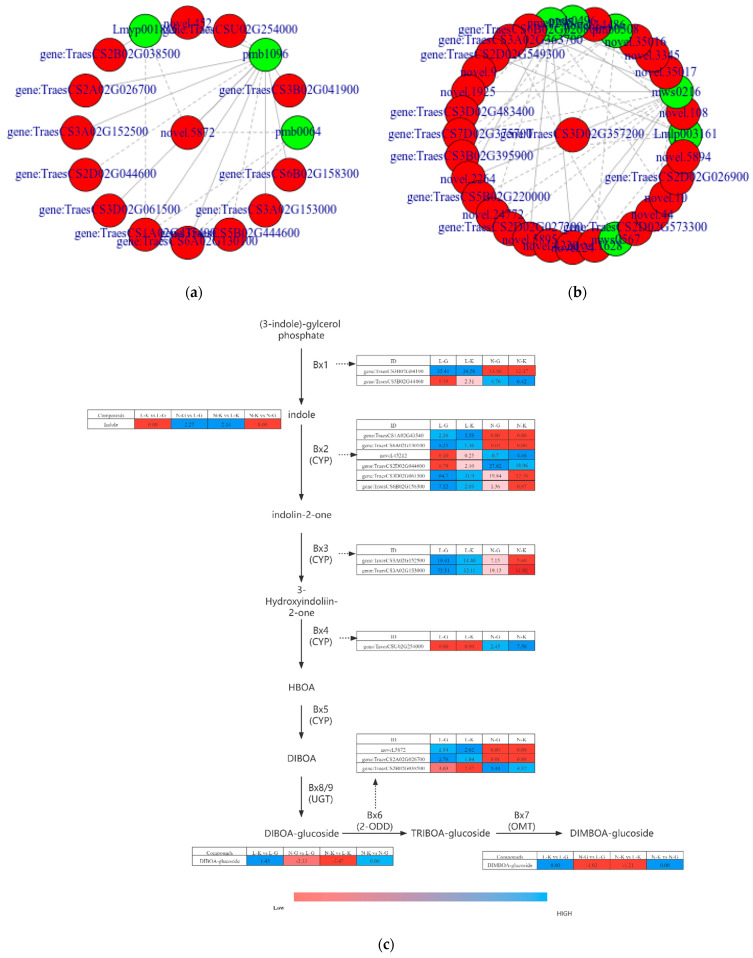
(**a**) Network diagram of differential genes and metabolites in Benzoxazinoid biosynthesis; (**b**) network diagram of differential genes and metabolites in arginine and proline metabolism; (**c**) biosynthesis mechanism of Benzoxazinoid in different combinations, blue and red colors represent the up-regulation and down-regulation of gene expression and metabolites, respectively.

To further explore the correlation between differential genes and metabolites in the two pathways after SB infection, we compared the difference between N-G and L-G and drew a network diagram to show the correlation between metabolites and genes. We then selected differential genes and metabolites with a correlation > 0.8 for the correlation network diagram (Figure 7a,b) and the mechanism diagram of relevant metabolic pathways (Figure 7c). Tables S2 and S3 show the Pearson correlation coefficients of related differential metabolites and genes for Benzoxazinoid biosynthesis and arginine and proline metabolism. In Benzoxazinoid biosynthesis, three different metabolites were involved: indole, DIBOA, and DIMBOA. The enzymes involved included indole-2-monooxygenase [EC:1.14.14.153], DIBOA-glucose dioxygenase BX6 [EC:1.14.20.2], peptide-methionine(R)-S-oxide reductase [EC:1.8.4.12], indole-2-one monooxygenase [EC:1.14.14.157], 3-hydroxyindole-2-one monooxygenase [EC: 1.14.14.109], DIBOA-glucose dioxygenase BX6 [EC:1.14.11.59], indole-3-glycerophosphate lyase [EC:4.1.2.8], tryptophan synthase [EC:4.1.2.8], and indole-2-monooxygenase [EC: 1.14.153]. Using correlation analysis, we found that *gene:TraesCS2A02G026700*, *novel.452*, *gene:TraesCS1A02G435400*, and *gene:TraesCSU02G254000* had a high correlation with indole (|PPC| > 0.9), among which, *gene:TraesCSU02G254000* negatively regulated indole. *Novel.5872*, *gene:TraesCS1A02G435400,* and *Novel.452* were negatively regulated by DIBOA. The regulation of *gene:TraesCSU02G254000* and *gene:TraesCS2B02G038500* on DIBOA was positive. Three genes showed strong correlation with DIMBOA, namely *Novel.5872*, *gene:TraesCS1A02G435400*, and *gene:TraesCS6A02G130100*, all of which were negatively regulated. In conclusion, we found that the changes of the three different metabolites were affected by *gene:TraesCS1A02G435400* and *novel. 5872* regulation. In addition, the expression trend of *gene:TraesCSU02G254000* and *gene:TraesCS2B02G038500* was consistent with that of DIBOA, which increased significantly in N-G and N-K.

In arginine and proline metabolism, six metabolites showed a strong correlation with 27 genes. The specific correlation coefficients are shown in Supplementary Table S2. The metabolites included trans-4-hydroxy-l-proline*, 4-acetylaminobutyric acid, p-coumarinyl agmatine, ferulic putrescine, N-ferulic agmatine, and 4-guanylbutyric acid. Fourteen related enzymes, such as acetaldehyde dehydrogenase [EC:1.2.1.31 1.2.1.8 1.2.1.3], δ-1-pyrroline-5-carboxylic acid synthase [EC:2.7.2.11 1.2.1.41], specific protease 1 [EC: 3.4.22.68], pyrroline-5-carboxylic acid reductase [EC:1.5.1.2], polyamine oxidase [EC: 1.5.3.14 1.5.3.16 1.5.3.], and nitric oxide synthase, were identified. Among them, trans-4-hydroxy-l-proline* had a high correlation with *gene:TraesCS2D02G027200*, *gene:TraesCS5B02G22000*, *gene:TraesCS6B02G020800*, *gene:TraesCS3B02G395900*, and *Novel.14486*. Through action and δ- 1-pyrroline-5-carboxylic acid synthetase [EC: 2.7.2.11 1.2.1.41] and acetaldehyde dehydrogenase [EC: 1.2.1.31 1.2.1.8 1.2.1.3], we realized the negative regulation of three metabolites of ferulic putrescine, p-coumarinyl agmatine, and N-ferulic agmatine. 4-Guanylbutyric acid is only negatively regulated by *gene:TraesCS3A02G363700*.

## 3. Discussion

In this study, we aimed to explore the differences between *T. aestivum* ssp and normal plants after infection with SB and different parts of the same susceptible plant. We also explored the response mechanism of *T. aestivum* ssp against SB by widely targeting metabolome and transcriptome. Resistance to SB is a complex quantitative trait. We found that the main metabolites of susceptible and resistant varieties are alkaloids, phenolic acids, flavonoids, organic acids, amino acids and their derivatives, and terpenes. The same was true in the susceptible varieties’ diseased and non-diseased parts. When the SB pathogen invades, the relevant metabolites and genes will change significantly in the crop to resist pathogen attack and start the defense response. Flavonoids such as gallicatechin, kaempferol, and rhamnose are mainly accumulated in resistant varieties and are considered antibacterial agents and antioxidants [34,35,36,37]. 7-Methylnaringin and apigenin-7,4’- dimethyl ether inhibit fungal growth and are significantly upregulated at the susceptible site, caused by the activation of self-defense response in plants. Alkaloids mainly include indole, DIBOA, DIMBOA, and phenolic amines, such as N-acetylputrescine, ferulic putrescine, N-ferulic agmatine, and N-ferulic agmatine, and organic acids such as 4-acetylaminobutyric and 4-guanidine butyric acids. These secondary metabolites play an important role in plant growth and development and resist the threat of SB. The erosion of pathogenic bacteria leads to the leaves showing a patchy wilting state and the photosynthetic system is seriously damaged, severely affecting plant photosynthesis, which is significantly different from that of normal leaves. In the rich concentration of GO, the genes related to photosynthesis are significantly enriched, indicating differences in photosynthesis between susceptible and resistant strains, consistent with the results of phenotypic differences. More accurately, it reflects the accuracy and reliability of our data.

**Figure 8:**
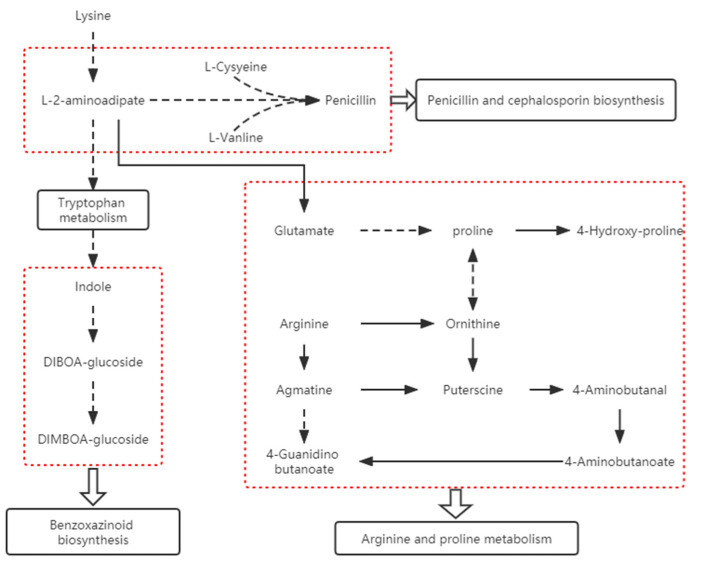
Links between metabolic pathways.

SB is considered to be a fungal disease. It is easy to breed pathogenic bacteria under high temperature and humidity conditions. In the fungal infection process, Benzoxazinoid biosynthesis is considered the second important manner to activate many gramineous crops [38]. BXs are released from the premise of constitutive storage after the microbial invasion. The antibacterial effect of BXs such as DIMBOA has been confirmed in corn, wheat, and other crops. As an important metabolite in the metabolic pathway of arginine and proline, proline can resist plant pathogens and abiotic factors [39,40,41,42]. It is considered to be a multifunctional amino acid and proline accumulates in the pathogen infection process [43]. Among differential metabolic pathways, penicillin and cephalosporin biosynthesis pathways were found to be most significant in our study. Penicillin is an antibacterial substance. The degradation of lysine leads to the increase of L-2 aminoadipic acid expression. L-cysteine and L-valine are highly expressed in susceptible strains caused by the self-defense effect in the early stage of infection, which lays the foundation for penicillin synthesis. L-2 aminoadipic acid—the degradation product of lysine—can be transformed into glutamate to produce proline and induce tryptophan metabolism. Tryptophan biosynthesis promotes indole synthesis and changes the expression of Benzoxazinoid biosynthesis-related metabolites. The connection of each channel is shown in Figure 8. We speculate that this is a metabolic pathway of Triticale infected with leaf blight, which first affects the metabolic activities of lysine, the degradation and metabolism of lysine, and then causes the changes in L-2 aminoadipic acid, resulting in the changes in the penicillin and cephalosporin biosynthesis, tryptophan metabolism, arginine and proline metabolism, and Benzoxazinoid biosynthesis.

We speculated that the biosynthesis of Benzoxazinoid is the key pathway of resistance to SB in iron husk wheat. In the study results, we found that indole, a metabolite related to the biosynthesis of Benzoxazinoid, was highly expressed in susceptible varieties. Concurrently, DIBOA and DIMBOA significantly accumulated in resistant varieties, consistent with the results of previous studies. Among them, *gene: TraesCS1A02G435400* and *Novel.5872*, which were strongly correlated with the three differential metabolites, had zero expression of FPKM in the resistant varieties, which might be caused by pathogens. We speculated that these two genes were only expressed when SB infected plants. *Gene:TraesCSU02G254000* and *gene:TraesCS2B02G038500* are highly correlated with DIBOA and are positively regulated. We speculated that *gene:TraesCSU02G254000* and *gene:TraesCS2B02G038500* are two key genes essential for resistance to SB.

In addition, arginine and proline metabolism were significantly enriched in the susceptible and normal positions of the same variety and the susceptible and resistant strains. This might be due to the self-defense effect of iron shell wheat in response to the invasion of pathogens. Trans-4-hydroxy-l-proline*, a proline derivative, is a rare sub amino acid known as an osmotic protector and antioxidant. It contributes to anti-tumor and synthetic antibiotics and accumulates significantly in the early stage of infection [44]. Phenolic amine is a special metabolite and plays an important role in plant biological disease resistance [45]. In arginine and proline metabolism, arginine can synthesize putrescine through two mechanisms, from ornithine to putrescine under ODC1 (ornithine decarboxylase [EC:4.1.1.17]) and from guanidine butylamine. Studies have shown that the high expression of odc1 is conducive to improving the resistance of rice-to-rice blast [46]. In our study, the annotated phenolic amine differential metabolites related to arginine and proline generation are derivatives of putrescine and agmatine, mainly n-acetylputrescine, p-coumarinyl putrescine, ferulic putrescine, p-coumarinyl agmatine, and n-ferulic agmatine. Through the correlation analysis of differential metabolites and genes, we found that gene: *TraesCS3B02G395900* and *novel.14486* are highly correlated with n-ferulic agmatine butylamine, p-coumaric agmatine butylamine, and ferulic putrescine, and are all negatively correlated. We speculate that arginine and proline metabolism changes are part of the plant defense mechanism and metabolite changes can be attributed to the resistance of plants to the invasion of pathogens.

Through this study we aim to evaluate the characteristic of iron shell wheat resource disease resistance and excellent resistance genes, for the wheat SB resistance provides new insight into some of the related mechanism, and the biological control of wheat leaf blight research provides materials and basis and broadens the SB resistant wheat varieties breeding, development, and utilization of the direction.

## 4. Materials and Methods

### 4.1. Materials

**Figure 9:**
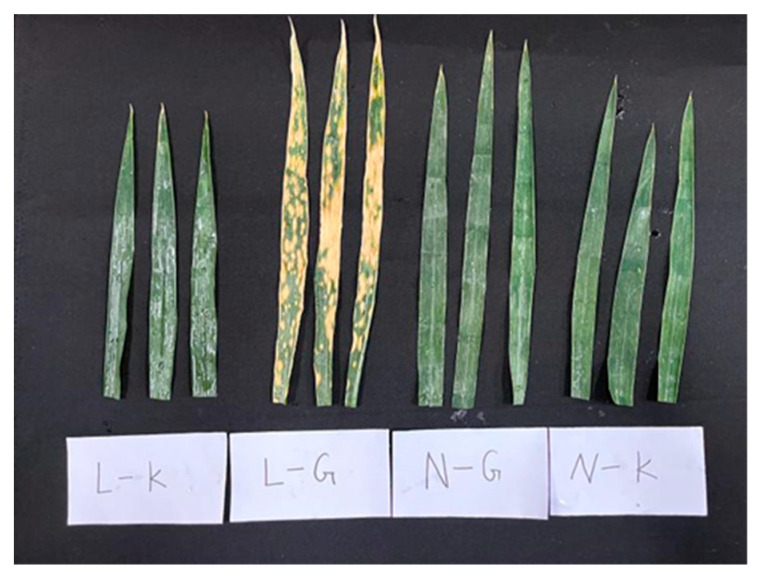
Four groups of leaf samples with three repetitions.

Fifty *Triticum aestivum* ssp. *yunnanense* were planted in a greenhouse in the XunDian Daheqiao experimental base of Yunnan Agricultural University (25°31′3″ N, 103°16′42″ E). At the booting stage, the plants were infected with SB in the greenhouse. As a result, Yunmai 0608, a susceptible variety, was identified as L, and Zhenkang iron shell wheat (Yunmai 0030), a resistant variety, was identified as N. In Lincang iron shell wheat (Yunmai 0608), a serious infection was observed in the second leaf of the plant, with no infection in the flag leaf. Therefore, the second leaf was marked as G and the flag leaf was marked as K. There were four groups of samples with three repetitions, namely L-G, L-K, N-G, and N-K (as shown in Figure 9). The cut samples were immediately placed in liquid nitrogen and in the refrigerator at −80 °C for standby. Then, the samples were sent to Wuhan Maiwei metabolism company for metabolome and transcriptome sequencing.

### 4.2. Widely Targeted Metabolome Detection and Analysis

The sample was placed in a freeze dryer (scientz-100f) for vacuum freeze drying and ground (30 Hz, 1.5 min) to powder using a zirconia bead grinder (mm 400, Retsch). Subsequently, 100 mg of powder was weighed and dissolved in 1.2 mL of 70% methanol extract, vortexed once every 30 min for 30 s for a total of 6 times and placed in a refrigerator at 4 °C overnight. Following centrifugation at 12,000 rpm for 10 min, the supernatant was absorbed using a microporous filter membrane (scaa-104, 0.22 μM aperture; Shanghai, China, http://www.anpel.com.cn/, accessed on 30 May 2021). The sample was filtered for analysis by ultra-performance liquid chromatography (UPLC) (Shimadzu nexera X2 https://www.shimadzu.com.cn/, accessed on 2 June 2021) Qtrap and tandem mass spectrometry (MS/MS) (Applied Biosystems 4500).

The chromatographic column for the liquid phase was an Agilent sb-c18, 1.8 µm, 2.1 mm * 100 mm. In the mobile phase, phase A was ultrapure water to which 0.1% formic acid was added and phase B was acetonitrile to which 0.1% formic acid was added. The proportion of phase B in the elution gradient was 5% at 0.00 min and the proportion of phase B increased linearly to 95% within 9.00 min and was maintained at 95% for 1 min. Subsequently, the composition of 95% A and 5.0% B was adjusted within 1.10 min and maintained for 2.9 min. The injection volume was 4 μL, with the flow rate set to 0.35 mL/min, and the temperature of the column oven set to 40 °C. The effluent was alternately connected to an ESI triple quadrupole linear ion trap (Qtrap). After the mass spectrum analysis data of metabolites in different samples were obtained through multiple reaction monitoring (MRM) analysis of triple quadrupole mass spectrometry, the mass spectrum peaks of all substances were integrated and the mass spectrum peaks of the same metabolite present in different samples were calibrated. The data were then analyzed qualitatively and quantitatively. Unsupervised principal component analysis (PCA) was performed using the statistical function prcomp in R (www.r-project.org, accessed on 5 June 2021). The correlation between the cluster coefficients of PCA and Pearson function were analyzed using the hierarchical correlation diagram of HCC. VIP values were extracted from OPLS-DA results, which also contained score plots and permutation plots, and generated using R package MetaboAnalystR. The data was log transformed (log2) and mean centered prior to OPLS-DA. To avoid overfitting, a permutation test (200 permutations) was performed. The subsequently identified metabolites were annotated using the KEGG Compound database (http://www.kegg.jp/kegg/compound/, accessed on 15 June 2021). Annotated metabolites were then mapped to the KEGG Pathway database (http://www.kegg.jp/kegg/pathway.html, accessed on 20 June 2021). Pathways with significantly regulated metabolites mapped were then fed into MSEA (metabolite sets enrichment analysis) and their significance was determined using hypergeometric test’s *p*-values.

### 4.3. Transcriptome Sequencing and Analysis

RNA of biological samples was extracted and detected and a library of qualified RNA was constructed to sequence high-quality data. Original data filtering, adapter removal, and quality trimming were performed using fastp v 0.19.3 (Haplox, Shenzheng, China). When the content of N in any sequenced reads exceeded 10% of the base number of the reads, paired reads were removed. When the base number of low-quality (Q ≤ 20) in any sequencing read exceeded 50% of the base number of the read, paired reads were removed to obtain clean reads for all subsequent analysis. The reference genome and its annotation file were downloaded from the designated website and HISAT v2 1.0 (Johns Hopkins University, Baltimore, MD, USA) was used to construct the index and compare the clean reads to the reference genome. StringTie v 1.3.4d (Johns Hopkins University, Baltimore, MD, USA), which uses a network flow algorithm and optional de novo to splice transcripts, was used for new gene prediction. FeatureCounts v 1.6.2 calculated the gene comparison and the FPKM value of each gene according to the gene length. Differential expression analysis between the two groups was performed using DESeq 2 v1.22.1 (The European Molecular Biology Laboratory) and the *p*-value was corrected using the Benjamini–Hochberg method. The corrected *p*-value and |log2foldchange| were used as the threshold for significant difference expression. Enrichment analysis was conducted based on the hypergeometric test. For KEGG, the hypergeometric distribution test was carried out in the pathway unit, and for GO, it is based on the GO term.

### 4.4. Combined Analysis of Transcriptome and Metabolome

By analyzing the metabolome and transcriptome results, the differential metabolites and genes in the same group were mapped to the KEGG pathway map simultaneously and then the histogram was drawn according to the enrichment analysis results to show the enrichment degree of pathways. Correlation analysis was conducted on the genes and metabolites detected in each different group. The cor program in the R package was used to calculate the Pearson correlation coefficient of genes and metabolites. The gene metabolites with a Pearson correlation coefficient >0.8 in each group were selected as a network diagram to demonstrate the correlation between metabolites and genes. Then, the overall correlation between the two groups of indicators was reflected by the canonical correlation coefficient. All differential genes and metabolites were selected for this study to establish the O2PLS model. The variables with high correlation and weight in different data were preliminarily judged through a load map and the important variables affecting other omics were selected.

### 4.5. RT-qPCR

Design7.9 design-specific primers were used for selecting genes. RT-qPCR was performed on a 96-well StepOnePlus instrument (Applied Biosystems, Foster City, CA, USA) according to the instructions of PerfectStart^TM^ SYBR Green qPCR SuperMix (TransGen Biotech, Beijing, China). The volume of the reaction system 20 µL, including 10 µL 2 × PerfectStart^TM^ SYBR Green qPCR Supermix, 0.4 µL calibration solution, 5.8 µL nuclease-free water, 0.4 µL of each primer (10 mm), and 3 µL cDNA (200 µg/µL). The thermal cycle was set for initial heat denaturation at 94 °C for 30 s, 94 °C for 5 s, and 40 cycles at 60 °C for 30 s. 26S rRNA was used as the internal control and the relative transcription levels were calculated using the was 2^−ΔΔCT^ method.

## 5. Conclusions

The resistance of Triticale to SB is an extremely complex quantitative trait, which is affected by various metabolic pathways. However, after excluding the inherent differences between varieties and the differences between different parts of the same plant, we conclude that the resistance of Triticale to SB is closely related to the Benzoxazinoid biosynthesis. Arginine and proline metabolism is essential for Triticale resistance to SB. The expression levels of *gene:TraesCSU02G254000* and *gene:TraesCS2B02G038500* contributed to the SB resistance of Triticale.

## Figures and Tables

**Table 1:** Types and quantities of metabolites.

Species of Metabolites	Number of Metabolites
amino acids and their derivatives	95
phenolic acids	178
nucleotides and their derivatives	67
flavone	234
lignans and coumarins	30
blending quality	3
alkaloid	111
terpenoids	22
organic acid	84
lipid	154
other	100
total:	1078

## Data Availability

Not applicable.

## Supplementary Materials

The following supporting information can be downloaded at: https://www.mdpi.com/article/10.3390/ijms23095184/s1.
