# Supplementary material for: Mechanisms of Resistance to Spot Blotch in Yunnan Iron Shell Wheat Based on Metabolome and Transcriptomics
Source: Int J Mol Sci. 2022 May 6;23(9):5184. doi: 10.3390/ijms23095184 (PMC9104156; doi:10.3390/ijms23095184)
Supplement: Supplementary file 1 [file ijms-23-05184-s001.zip › ijms-1691532-supplementary.pdf]

International Journal of
Molecular Sciences

MDPI

Article

Mechanisms of resistance to Septoria blotch in Yunnan iron shell wheat based on metabolome and transcritomics

Zhang Xuesong [1,†], Huang Tingzhi [1,†], Wang Qianchao[1], Guo Yirui[1], Zhang Ping[1],Xie Heng[1], Liu Junna[1] ,Li Li[1], Zhang Chuanli[2, *], Qin Peng[1,*]

[1]College of Agronomy and Biotechnology, Yunnan Agricultural University, Kunming 650201, China; 1271035784@qq.com (X.Z.); 1462757849@qq.com (T.H.); 2512078639@qq.com (Q.W.); 2430063338@qq.com (Y.G.); 1038621748@qq.com (P.Z.); 1143381995@qq.com (H.X.); 562701080@qq.com (J.L.); 1937137968@qq.com (L.L.)

[2] College of Tropical Crops, Yunnan Agricultural University Pu'er 665000, China; College of Agronomy and Biotechnology Yunnan Agricultural University Kunming 650201, China

[*] Correspondence: zhchuanli00@163.com, Tel.: 13769957693 (C.Z.); wheat-quinoa@ynau.edu.cn, Tel.:13508806942 (P.Q.)

[†] These authors have contributed equally to this work.

Supplementary Materials

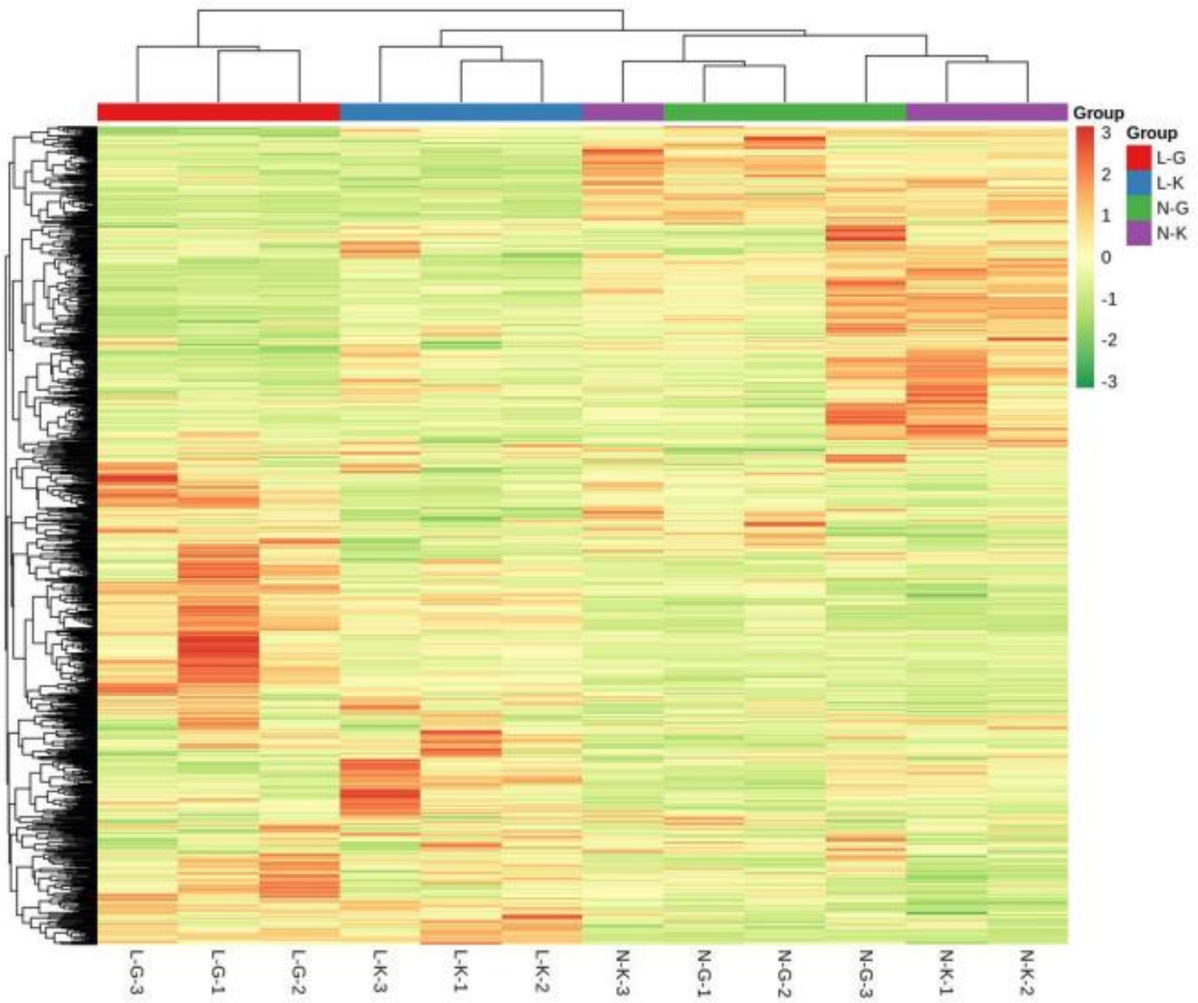

Figure S1. Sample longitudinal blood clustering heat map Note: the horizontal direction is the sample name, the vertical direction is the metabolite information, group is the grouping, class is the substance classification, and different colors are the values obtained after the standardization of relative content (red represents high content and green represents low cont.

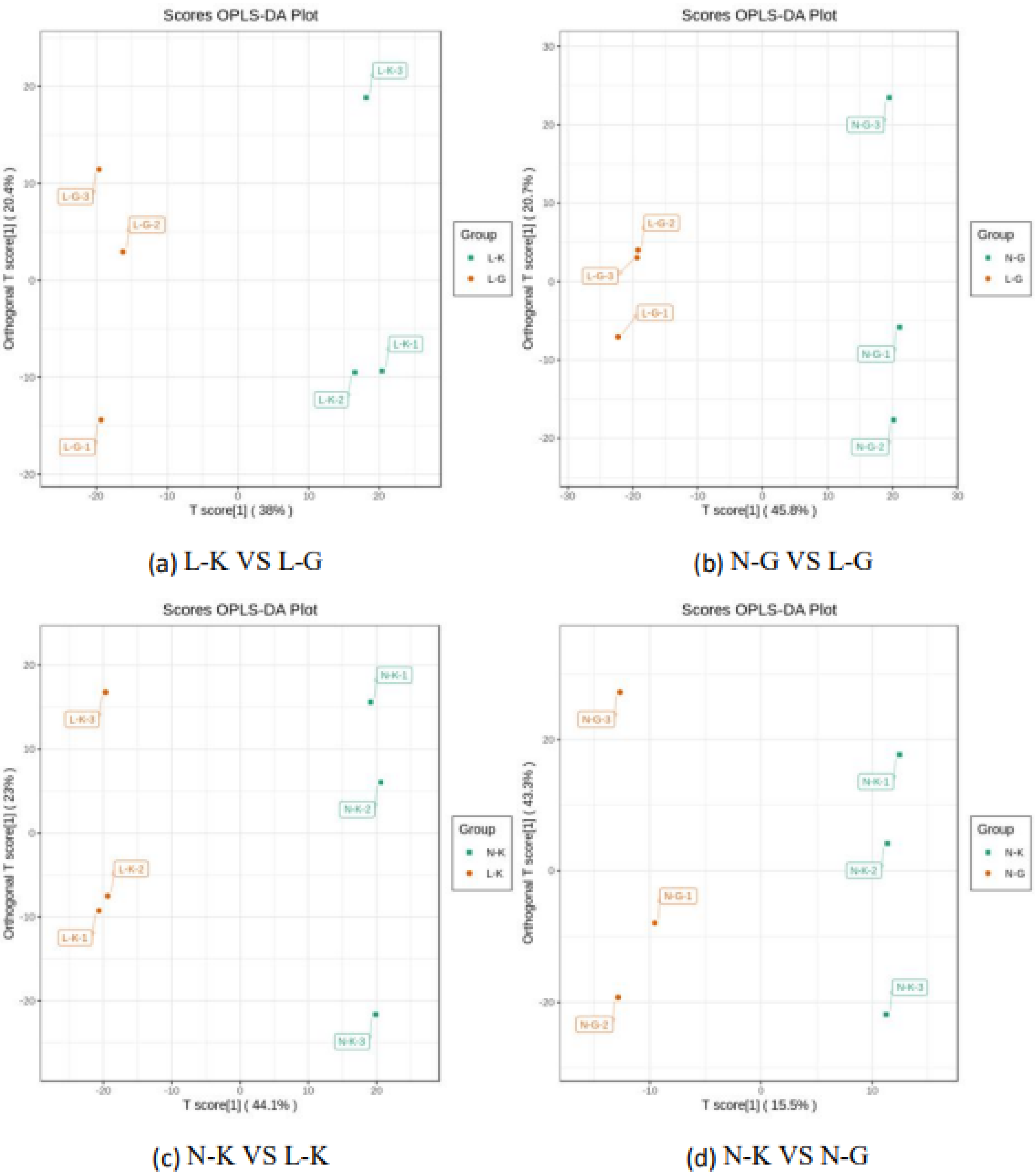

Figure S2. OPLS-DA analysis scores of different combined metabolic groups Note: the abscissa represents the predicted principal component, and the difference between groups can be seen in the abscissa direction; The ordinate represents the orthogonal principal component, and the difference within the group can be seen in the direction of the ordinate; The percentage represents the interpretation of the component to the data set. Each point in the figure represents a sample, samples in the same group are represented by the same color, and group is a group.

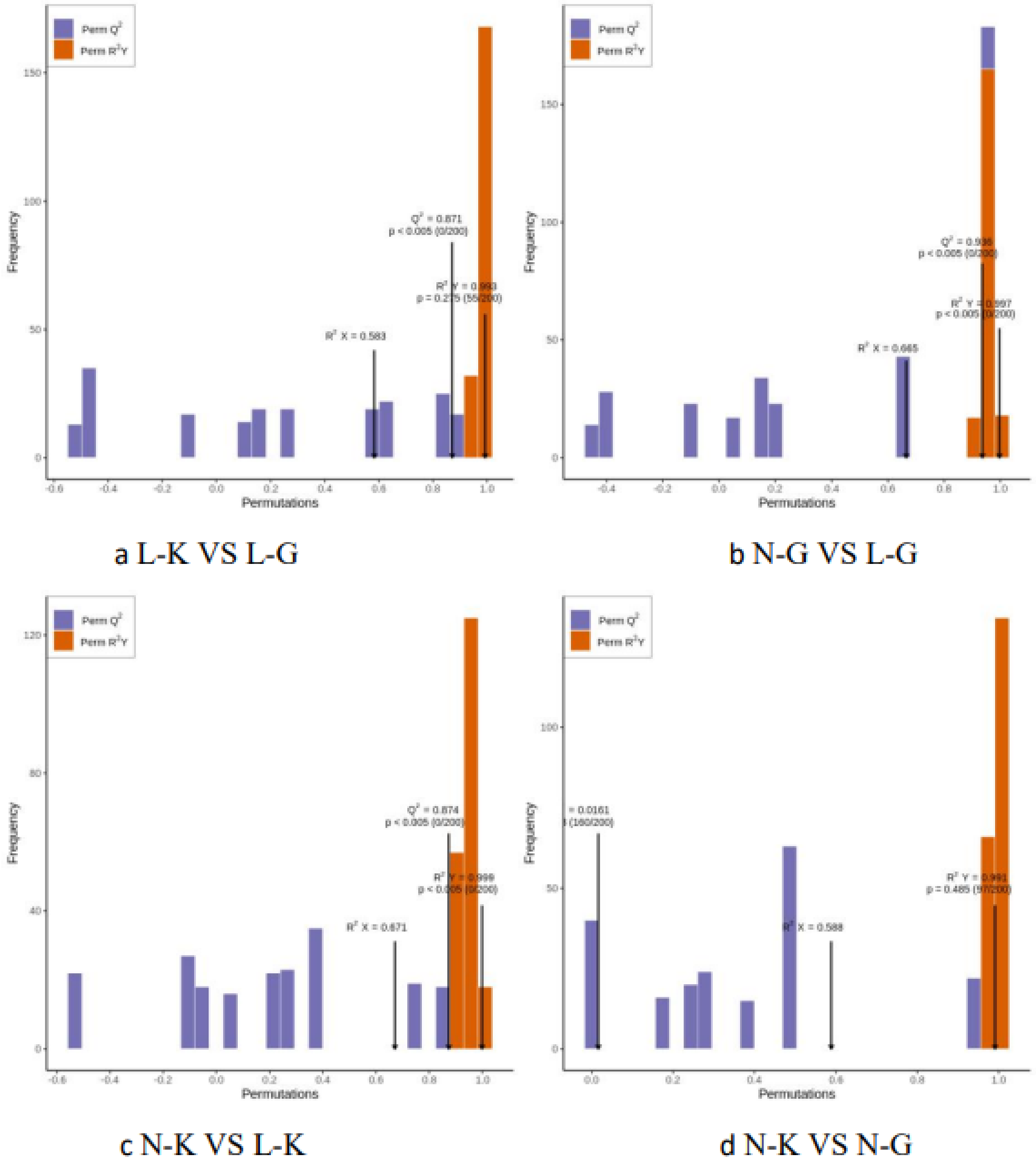

Figure S3. opls-da verification diagram.

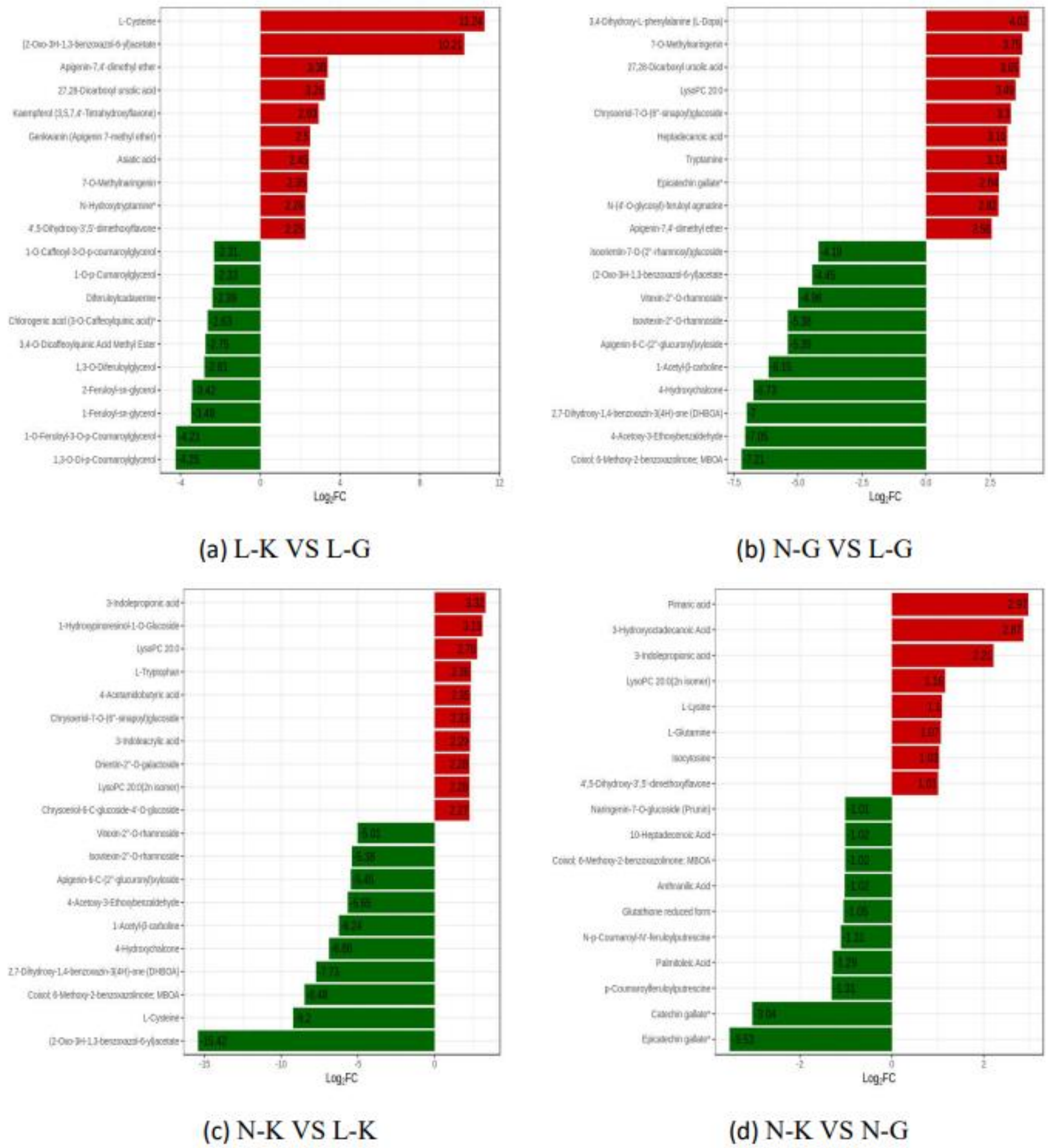

Figure S4. Difference analysis of different metabolic groups. (a), (b), and (c) refer to the top ten metabolites up and downregulated by each combination of differential metabolites. Red represents upregulated differential metabolites, and the green represents downregulated differential.

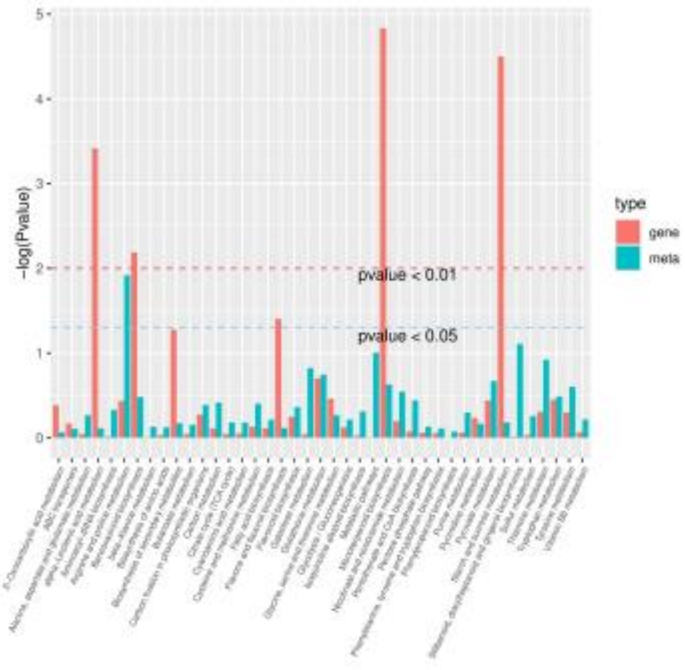

(a)L-K VS L-G

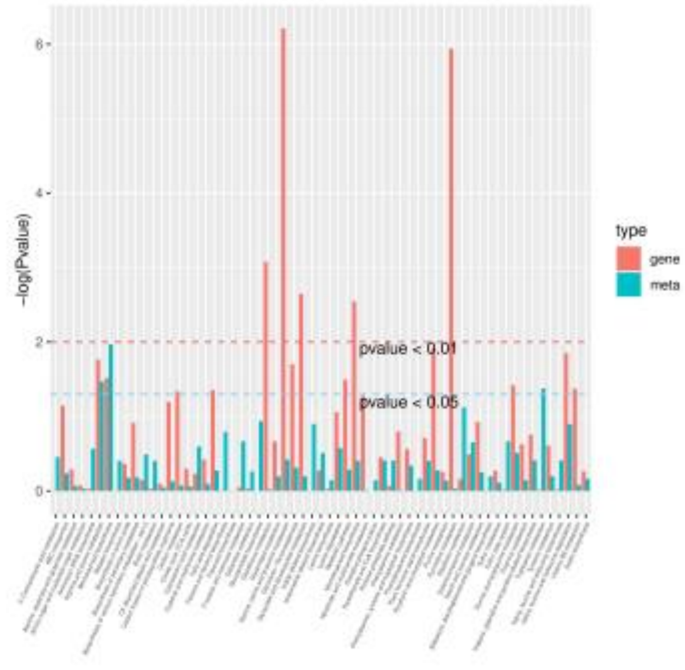

(b)N-G VS L-G

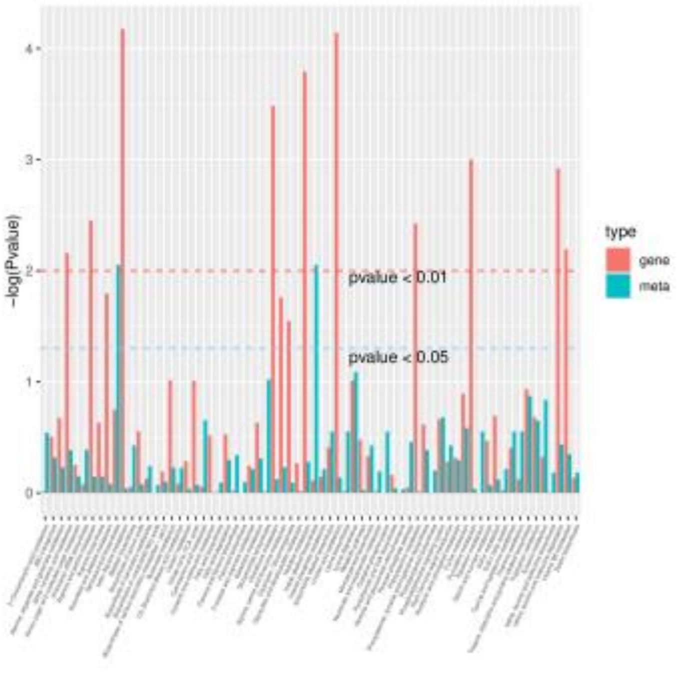

(c)N-K VS L-K

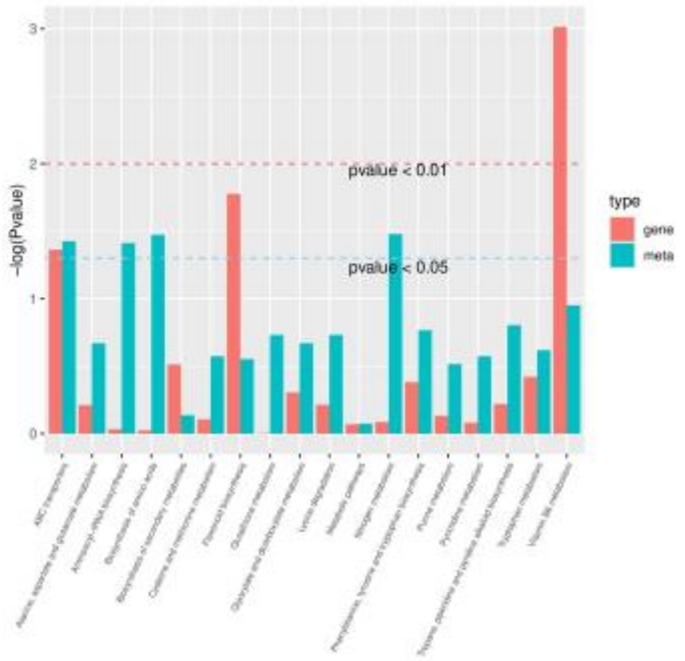

Figure S5. KEGG enrichment analysis pvalue histogram Note: the very coordinates of the histogram represent the metabolic pathway, the red in the ordinate represents the enriched pvalue value of differential genes, and the green represents the enriched pvalue value of differential metabolites, which is expressed by - log (p-value). The higher the ordinate, the stronger the enrichment degree.

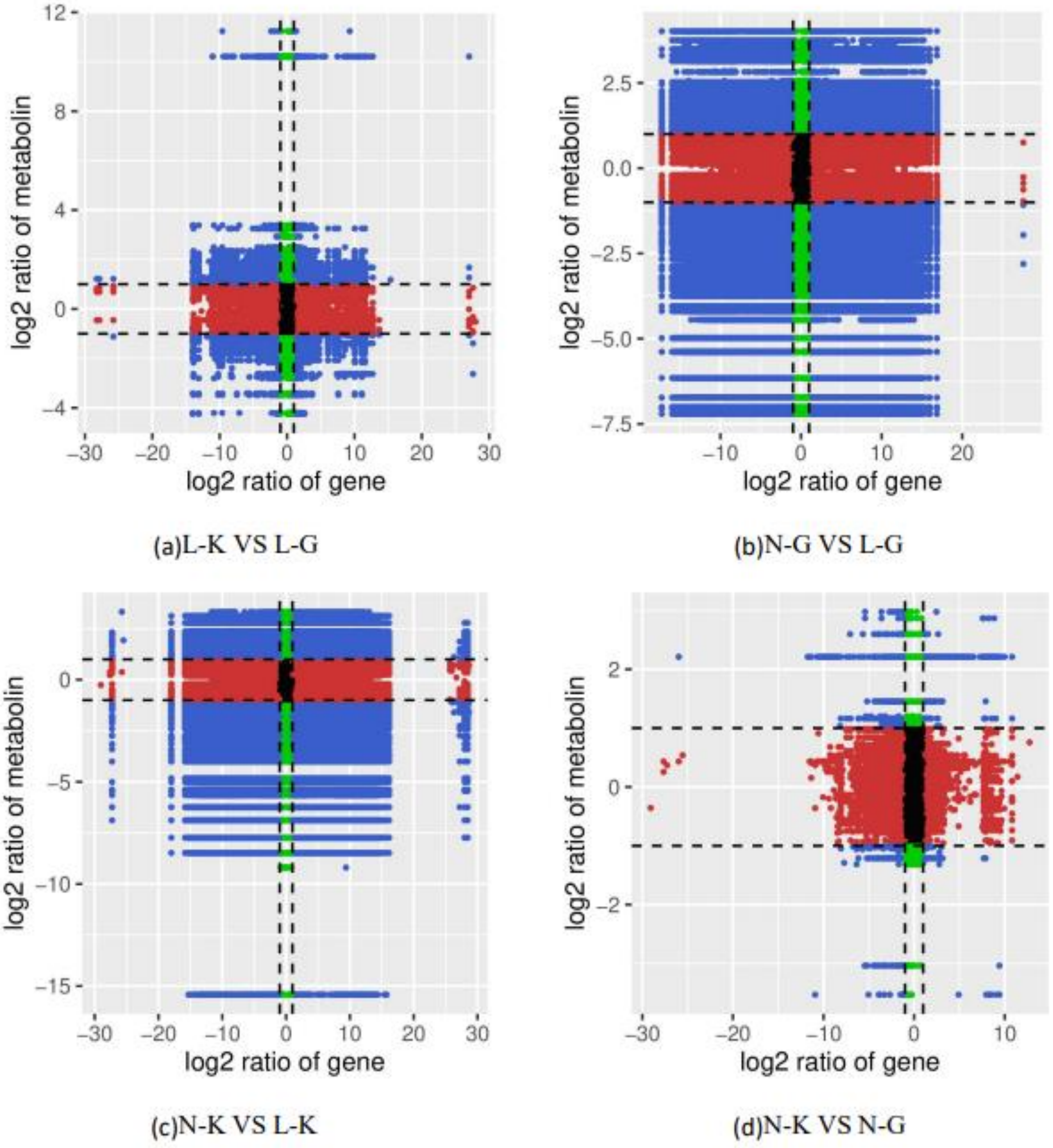

Figure S6. Nine quadrant diagram of correlation analysis Note: the nine quadrant chart shows the difference multiple of gene metabolites with Pearson correlation coefficient greater than 0.8 in each difference group. It is divided into 1-9 quadrants from left to right and from top to bottom with black line.

Table S1: K-means analysis of heterometabolites

Primary classification of substances	Sub Class 1	Sub Class 2	Sub Class 3	Sub Class 4	Sub Class 5	Sub Class 6	Sub Class 7	Sub Class 8	Sub Class 9
Amino acids and their derivatives	0	1	3	1	1	2	8	5	17
Phenolic acids	9	4	6	8	2	14	4	20	9
Nucleotides and their derivatives	4	2	0	4	1	2	5	0	7
Flavone	7	12	7	18	4	24	11	0	8
Lignans and coumarins	3	2	1	0	0	4	1	5	1
Other classes	6	0	7	1	1	7	8	4	4
Blending quality	0	0	0	0	0	1	0	1	1
Alkaloid	6	5	5	8	3	8	17	2	19
Terpenoids	0	1	1	1	1	3	5	0	2
Organic acid	4	1	1	3	3	3	3	2	7
Lipid	11	1	1	2	1	7	4	1	6

Table.S2: association analysis of differential metabolites and differential genes in Benzoxazinoid biosynthesis

Gene Name	KEGG	Compound	PCC
novel.5872	K13229	DIMBOA glucoside	-0.829
gene:TraesCS3B02G041900	K13222	Indole	0.867
gene:TraesCSU02G254000	K13225	Indole	-0.9
novel.452	K07305	Indole	0.905
gene:TraesCSU02G254000	K13225	DIBOA glucose	0.809
novel.5872	K13229	DIBOA glucose	-0.868
gene:TraesCS2B02G038500	K13229	DIBOA glucose	0.848
gene:TraesCS2A02G026700	K13229	Indole	0.95
novel.452	K07305	DIBOA glucose	-0.812
gene:TraesCS3A02G152500	K13224	Indole	0.83
gene:TraesCS2D02G044600	K13223	Indole	-0.864
gene:TraesCS3D02G061500	K13223	Indole	0.805
gene:TraesCS1A02G435400	K13223	DIMBOA glucoside	-0.814
gene:TraesCS1A02G435400	K13223	DIBOA glucose	-0.864
novel.5872	K13229	Indole	0.885
gene:TraesCS6A02G130100	K13223	Indole	0.801
gene:TraesCS1A02G435400	K13223	Indole	0.901
gene:TraesCS5B02G444600	K13222	Indole	-0.893
gene:TraesCS6A02G130100	K13223	DIMBOA glucoside	-0.815
gene:TraesCS3A02G153000	K13224	Indole	0.815
gene:TraesCS6B02G158300	K13223	Indole	0.837

Table.S3: association analysis of differential metabolites and differential genes in arginine and proline metabolism

Gene Name	KEGG	Compound	PCC
gene:TraesCS3D02G357200	K12657	N-Feruloylputrescine	-0.876
novel.108	K08592	Trans-4-Hydroxy-L-proline*	0.816
novel.35017	K14085	Trans-4-Hydroxy-L-proline*	0.819
novel.3345	K14085	Trans-4-Hydroxy-L-proline*	0.816
novel.35016	K14085	p-Coumaroylagmatine	0.82
novel.14486	K14085	N-Feruloylagmatine	-0.813
novel.108	K08592	p-Coumaroylagmatine	0.808
novel.108	K08592	4-Acetamidobutyric acid	0.82
gene:TraesCS6B02G020800	K13427	Trans-4-Hydroxy-L-proline*	0.84
gene:TraesCS3A02G363700	K12657	N-Feruloylputrescine	-0.805
gene:TraesCS2D02G549300	K17839	p-Coumaroylagmatine	0.823
novel.9	--	p-Coumaroylagmatine	0.813
gene:TraesCS3D02G357200	K12657	p-Coumaroylagmatine	-0.894
novel.1925	K17086	Trans-4-Hydroxy-L-proline*	0.808
gene:TraesCS3D02G483400	K00286	4-Acetamidobutyric acid	-0.842
gene:TraesCS7D02G375700	K13366	Trans-4-Hydroxy-L-proline*	-0.825
gene:TraesCS3B02G395900	K12657	N-Feruloylagmatine	-0.809
novel.2264	K00286	4-Acetamidobutyric acid	0.829
gene:TraesCS5B02G220000	K01611	Trans-4-Hydroxy-L-proline*	-0.831
novel.24772	K14085	4-Acetamidobutyric acid	-0.811
novel.35017	K14085	p-Coumaroylagmatine	0.807
novel.35016	K14085	Trans-4-Hydroxy-L-proline*	0.821
gene:TraesCS6B02G020800	K13427	4-Acetamidobutyric acid	0.816
gene:TraesCS2D02G027200	K01426	Trans-4-Hydroxy-L-proline*	-0.865
novel.14486	K14085	p-Coumaroylagmatine	-0.876
novel.3345	K14085	4-Acetamidobutyric acid	0.832

46

novel.5895	K14085	4-Acetamidobutyric acid	0.813
novel.4220	K14085	Trans-4-Hydroxy-L-proline*	0.815
novel.24	--	4-Acetamidobutyric acid	0.817
novel.1628	--	N-Feruloylputrescine	-0.853
novel.24	--	Trans-4-Hydroxy-L-proline*	0.818
novel.35016	K14085	4-Acetamidobutyric acid	0.805
gene:TraesCS3A02G363700	K12657	4-Guanidinobutyric acid	-0.807
gene:TraesCS2D02G573300	K01426	N-Feruloylputrescine	0.805
novel.24772	K14085	Trans-4-Hydroxy-L-proline*	-0.81
novel.1925	K17086	4-Acetamidobutyric acid	0.818
gene:TraesCS3A02G363700	K12657	p-Coumaroylagmatine	-0.815
novel.1628	--	p-Coumaroylagmatine	-0.867
gene:TraesCS2D02G549300	K17839	N-Feruloylputrescine	0.82
novel.14486	K14085	N-Feruloylputrescine	-0.855
novel.44	K12862	Trans-4-Hydroxy-L-proline*	-0.811
gene:TraesCS3B02G395900	K12657	N-Feruloylputrescine	-0.893
novel.10	--	4-Acetamidobutyric acid	-0.82
novel.2264	K00286	Trans-4-Hydroxy-L-proline*	0.808
novel.35017	K14085	4-Acetamidobutyric acid	0.814
gene:TraesCS2D02G026900	K01426	Trans-4-Hydroxy-L-proline*	-0.815
gene:TraesCS3B02G395900	K12657	p-Coumaroylagmatine	-0.897
novel.5894	K14085	Trans-4-Hydroxy-L-proline*	0.802
gene:TraesCS7D02G375700	K13366	p-Coumaroylagmatine	-0.803
